# Exploring Energy Conservation in Sulphate‐Dependent Anaerobic Methane‐Oxidising Consortia Through Metabolic Modelling

**DOI:** 10.1111/1462-2920.70156

**Published:** 2025-07-24

**Authors:** Gordon Bowman, Zena Jensvold, Qusheng Jin

**Affiliations:** ^1^ Geobiology Group University of Oregon Eugene Oregon USA

## Abstract

Anaerobic oxidation of methane (AOM) coupled with sulphate reduction (SR) is a crucial microbial process that mitigates methane emissions, a major contributor to climate change. However, the bioenergetics underlying this process remains poorly understood. Here, we present a metabolic model to quantify energy fluxes and conservation in AOM consortia by integrating enzyme‐level thermodynamics and kinetics. Unlike previous models that impose artificial constraints on energy conservation kinetics and efficiency, our approach mechanistically predicts ATP yields and energy efficiencies. We show that both anaerobic methanotrophic archaea (ANME) and sulphate‐reducing bacteria (SRB) invest energy in substrate activation, synthesising ATP with comparable yields (0.23–0.24 mol ATP per mol methane or sulphate), while achieving remarkable thermodynamic efficiency (~60%). However, ANME exhibit a higher return on investment (ROI, 18%) than SRB (11%) due to more efficient substrate activation. These findings highlight fundamental bioenergetic constraints governing methane oxidation and SR in anoxic environments, enhancing our understanding of how microbial processes regulate methane fluxes in natural ecosystems. By providing a quantitative framework for microbial energy conservation, our study advances biogeochemical modelling and informs strategies for methane mitigation in marine sediments and other anaerobic environments critical to climate regulation.

## Graphical abstract

This study presents a metabolic model to quantify energy conservation in sulphate‐dependent anaerobic methane oxidation. Results reveal that anaerobic methanotrophic archaea and sulphate‐reducing bacteria exhibit similar ATP yields (Y_ATP_), achieving high thermodynamic efficiency (*η*) but distinct returns on investment (ROI), providing key insights into microbial methane mitigation and biogeochemical modelling.

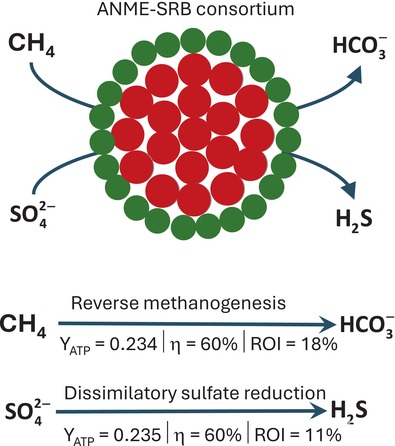

## 1 Introduction

Methane is a potent greenhouse gas with a global warming potential 25 times greater than that of carbon dioxide over a 100‐year period (IPCC 2006). Microbial processes, particularly anaerobic oxidation of methane (AOM), help mitigate methane emissions and stabilise Earth's climate. AOM coupled with sulphate reduction (SR) is a major methane sink in anoxic environments, removing ~382 Tg of methane annually (Reeburgh 2007; Gao et al. 2022). Despite its significance, the bioenergetics of AOM‐SR remains poorly understood, especially regarding how microorganisms catalyse these reactions and conserve energy under extreme thermodynamic constraints.

AOM‐SR is mediated by syntrophic consortia of anaerobic methanotrophic archaea (ANME) and sulphate‐reducing bacteria (SRB) (Knittel and Boetius 2009; Bhattarai et al. 2019). These consortia operate near thermodynamic equilibrium, with energy yields ranging from −10 to −40 kJ per mol of methane (Table 1) (Knittel and Boetius 2009). Such limited energy yields necessitate highly efficient energy conservation strategies to sustain metabolism. Understanding these strategies is critical for addressing knowledge gaps in AOM‐SR bioenergetics and predicting the role of these consortia in methane cycling (Dale et al. 2006; Knab et al. 2008).

**TABLE 1:** Thermodynamic, bioenergetic and kinetic constraints on sulphate‐dependent AOM consortia.^a^

Reaction	Reaction equation^b^	Reaction thermodynamics			Energetic yield					Kinetic limitation		
		*E*_A_ (mV)	*E*_D_ (mV)	Δ*G* (kJ·mol^−1^)	Δ*ψ* (V)	*ν*_+_	*Y*_ATP_ ^c^	*η* (%)^d^	ROI (%)^e^	*f* (kJ·mol^−1^)^f^	*χ*	*F*_T_
AOM‐SR	${\mathrm{CH}}_{4}\left(e\right)+{\mathrm{SO}}_{4}^{2-}\left(e\right)+H^{+}={\mathrm{HCO}}_{3}^{-}\left(e\right)+H_{2}S\left(e\right)+H_{2}O$	−209.6	−259.6	−38.6	—	—	0.469	59.4	—	—	—	—
Reverse methanogenesis	${CH_{4}\left(e\right)+3\;H_{2}O+4\;\mathrm{MP}=9\;H^{+}+\mathrm{HCO}}_{3}^{-}\left(e\right)+4\;\mathrm{MP}H_{2}$	−236.0	−259.6	−18.2	206.4	0.70	0.234	62.7	18	4.3	2.46	0.51
Sulphate reduction	${\mathrm{SO}}_{4}^{2-}\left(e\right)+10\;H^{+}+8\;{\text{TpIc}}_{\mathrm{red}}=H_{2}S\left(e\right)+4\;H_{2}O+8\;{\text{TpIc}}_{\mathrm{ox}}$	−209.6	−236.0	−20.4	129.2	0.94^g^	0.235	56.5	11	8.6	2.01	0.84

*Note:* The table summarises the stoichiometric equations and the parameters associated with reaction thermodynamics, including redox potentials of electron acceptors (*E*_A_) and donors (*E*_D_) and Gibbs free energy change (Δ*G*). It also highlights energetic yields, such as membrane potential (Δ*ψ*), ion translocation stoichiometry *ν*_+_, ATP yield *Y*_ATP_, thermodynamic efficiency *η* and return on investment (ROI). Kinetic limitations are described by the thermodynamic drive *f* for catabolism, average stoichiometric number *χ*, and thermodynamic potential factor *F*_T_. These parameters are presented for the overall sulphate‐dependent anaerobic methane oxidation (AOM‐SR), reverse methanogenesis by ANME, and sulphate reduction by SRB under typical laboratory bioreactor conditions.

^a^ The bioreactor is assumed to maintain a pH of 7 and contain 10 mM methane, 10 mM bicarbonate, 10 mM sulphate and 1 mM sulphide.

^b^ (e) represents metabolites in the environment.

^c^ Due to differences in membrane potentials, ATP synthases in ANME and SRB synthesise one ATP by translocating inward across the membrane 3 and 4 protons, respectively.

^d^ The fraction of total energy allocated to ANME or SRB that is conserved in ATP synthesis, calculated with a phosphorylation energy of 48.9 kJ⋅(mol ATP)^−1^, determined from the assumed concentrations of ATP, ADP and Pi.

^e^ ROI is the ratio of the energy conserved by ATP synthesis to the energy invested in substrate activation.

^f^ Thermodynamic drive *f* is calculated as the difference between the energy allocated to ANME (or SRB) and the energy conserved via ion translocation.

^g^ The net number of charges per reaction is calculated as the total charges translocated outward across the membrane minus the charges translocated inward by SULP for sulphate accumulation and 8 charges consumed in the synthesis of 2 ATP molecules required for sulphate activation to APS.

Culturing ANME‐SRB consortia is challenging (Bhattarai et al. 2019), making metabolic modelling a powerful tool for studying these microbial partnerships (Jin 2023). Previous models have described nitrate‐dependent AOM using stoichiometric metabolic modelling (Arshad et al. 2015; He et al. 2022), and tracked isotope fractionation in ANME via kinetic modelling (Wegener et al. 2021). However, existing sulphate‐dependent AOM models lack enzyme‐specific kinetics and thermodynamics, as well as adequate representations of ANME‐SRB interactions, limiting their ability to predict energy conservation strategies. Additionally, many models impose artificial constraints on syntrophic kinetics and metabolic efficiency, further reducing their predictive power (Jin 2023).

To address these limitations, we developed a thermodynamically constrained kinetic metabolic model for sulphate‐dependent AOM consortia. Our model focuses on how ANME and SRB conserve energy from AOM‐SR reactions. Beyond the established pathways of reverse methanogenesis and dissimilatory SR (Wang et al. 2014; Pereira et al. 2011; McGlynn et al. 2015; Wegener et al. 2015), our model aims to provide ab initio predictions of ATP yields, thermodynamic efficiencies and returns on investment (ROI) in energy metabolism. These predictions are based on tractable enzyme parameters and assumptions about syntrophic interactions, without imposing artificial constraints on microbial kinetics or metabolic efficiency.

Our model integrates syntrophic interactions, catabolic pathways and enzyme thermodynamics and kinetics. At its core, it explicitly represents enzyme thermodynamics and kinetics, with these constraints propagating through entire catabolic pathways, capturing the impact of low‐energy yields characteristic of AOM‐SR reactions (Dale et al. 2006; Knab et al. 2008). Notably, enzyme thermodynamics and kinetics are shaped not only by AOM‐SR energy yields but also by pathway structure and syntrophic interactions (Fell 1997; Plaxton 2004; Moreno‐Sánchez et al. 2008). By incorporating these factors, the model advances prior computational studies of AOM (Dale et al. 2006; He et al. 2019, 2021; Orcutt and Meile 2008) and provides a mechanistic foundation for understanding microbial energy metabolism in methane‐rich anoxic environments.

In this study, we apply our metabolic model to laboratory bioreactor conditions to address three fundamental questions:

- How do ANME overcome the thermodynamic barrier of methane activation?
- What mechanisms enable AOM consortia to conserve energy and synthesise ATP?
- To what extent do thermodynamic constraints regulate methane oxidation and SR kinetics?

By answering these questions, our study provides new insights into the energy conservation strategies of ANME and SRB, revealing how these microorganisms sustain metabolism under extreme thermodynamic constraints. Quantifying these processes enhances our understanding of microbial adaptation in methane‐rich anoxic environments and informs strategies for methane mitigation.

## 2 Methods

### 2.1 Model Overview

**FIGURE 1:**
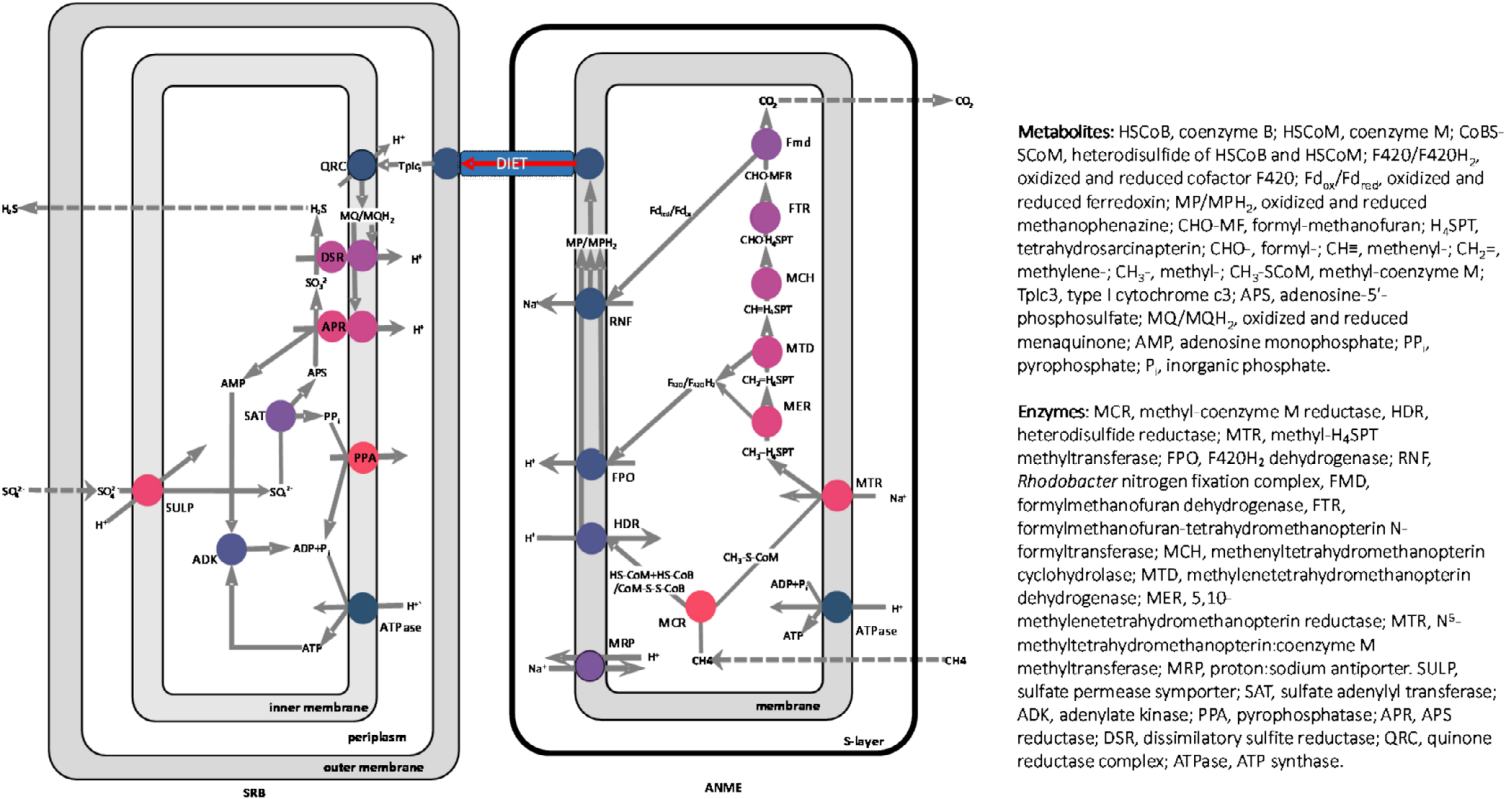
Energy metabolism in the ANME/SRB consortium. The sulphate reduction network follows the classical dissimilatory sulphate reduction pathway (Pereira et al. 2011); the reverse methanogenesis pathway is modelled based on findings related to sulphate‐dependent ANME‐1 and ANME‐2, as well as nitrate‐dependent ANME‐2d (Arshad et al. 2015; He et al. 2022; Wang et al. 2014; McGlynn et al. 2015); these two networks are interconnected via direct interspecies electron transfer (DIET) (McGlynn et al. 2015; Wegener et al. 2015).

Our metabolic model consists of three interconnected compartments: ANME cells, SRB cells and the surrounding environment (Figure 1). The environmental compartment contains methane, sulphate, bicarbonate and sulphide, which exchange with microbial cells via diffusion. ANME and SRB are linked via direct interspecies electron transfer (DIET) to couple methane oxidation and SR (McGlynn et al. 2015; Wegener et al. 2015). Each microbial compartment is further divided into cytoplasmic and membrane regions to model chemiosmotic ion translocation and membrane potential.

Each microbial compartment houses a kinetic submodel for reverse methanogenesis or dissimilatory SR. The submodels are initial value problems (IVPs) that consist of systems of ordinary differential equations (ODEs). Each ODE describes metabolite production or consumption as stoichiometric combinations of enzyme‐catalysed reaction rates and diffusion fluxes (see Supporting Information S1). Metabolic fluxes, expressed in mmol·g^−1^·d^−1^ (cell dry weight basis), represent community‐level activity within shell‐shaped ANME‐SRB aggregates.

Enzyme reactions follow reversible Michaelis–Menten kinetics, incorporating thermodynamic feedback to account for Gibbs free energy changes and chemiosmotic coupling (Jin et al. 2022). The free energy change Δ*G*_i_ of the reaction catalysed by enzyme i is calculated as:

(1) $$\Delta G_{i}=\mathrm{RT}\ln\left(\frac{Q_{i}}{K_{i}}\right)+\nu_{+,i}F\Delta \psi$$

where *R* is the gas constant (8.3145 J⋅mol^−1^⋅K^−1^), *T* is the absolute temperature (K), *Q*_i_ is the reaction quotient calculated from the concentrations of reactants and products, *K*_i_ is the equilibrium constant of the reaction (see Supporting Information S1), *ν*_+,i_ is the stoichiometric coefficient of protons or sodium cations translocated out of the cytoplasm in the reaction of enzyme i, F is the Faraday constant (96,485 C·mol^−1^) and Δ*ψ* is the membrane potential (V).

Enzyme kinetic parameters for SRB were sourced from axenic SRB, while those for ANME were adapted from methanogens (see Supporting Information S1 and S2). DIET follows gradient diffusion models (Desmond‐le et al. 2021), and diffusive fluxes are computed using Fick's law, considering aggregate geometry and cell density (see Supporting Information S3). Ion translocation and the resulting membrane potential Δ*ψ* are tracked using Faraday's law (Jin et al. 2022):

(2) $$\frac{d\Delta \psi }{\mathrm{dt}}=\frac{F}{C_{m}}\sum_{i}\nu_{+,i}\cdot \nu_{i}$$

where *C*_m_ is the membrane capacitance (C⋅V^−1^).

Cytoplasmic ATP, ADP and phosphate concentrations were set at 10, 1 and 10 mM, respectively, consistent with experimental measurements and established bioenergetic assumptions for methanogens (Santos et al. 1989; Thauer et al. 1977; Thauer 2011). Initial concentrations of all other metabolites, along with their data sources, are provided in Supporting Information S1.

### 2.2 Simulation Setup

We formulated a dual‐objective optimisation problem to estimate enzyme abundances and ion translocation stoichiometries. These parameters were treated as control variables to maximise the ATP production fluxes of ANME (*J*_RM,ATP_) and SRB (*J*_SR,ATP_).

(3) $$\begin{array}{l} \max\left(J_{\mathrm{ATP},\mathrm{RM}}\left(\phi_{\mathrm{RM},i},\nu_{\mathrm{RM},i+}\right),J_{\mathrm{ATP},\mathrm{SR}}\left(\phi_{\mathrm{SR},i},\nu_{\mathrm{SR},i+}\right)\right)\\ \begin{array}{cc} \text{subject to} & \phi_{\mathrm{RM},i}\in \left[0,\phi_{\mathrm{RM},\text{iMax}}\right],\phi_{\mathrm{SR},i}\in \left[0,\phi_{\mathrm{SR},\text{iMax}}\right]\\ & \nu_{\mathrm{RM},i+}\in \left[0,\nu_{+,\max}\right],\nu_{\mathrm{SR},i+}\in \left[0,\nu_{+,\max}\right] \end{array} \end{array}$$

where *ϕ*_RM,i_ and *ϕ*_SR,i_ represent the proteome fractions of enzymes in ANME and SRB, respectively, *ν*_RM,i+_ and *ν*_SR,i+_ are their stoichiometric coefficients of ion translocation, *ϕ*_RM,iMax_ and *ϕ*_SR,iMax_ are the upper bounds of the enzyme proteome fractions, and *ν*_+,max_ is the maximum possible number of charges translocated per reaction. For example, methyl coenzyme M reductase (MCR) is known to have a maximum proteome fraction of 12%, based on laboratory measurements of ANME under methane‐oxidising conditions (Krüger et al. 2003; Scheller et al. 2010; Ankel‐Fuchs et al. 1984). The optimisation was constrained by the ODEs of the kinetic model and hence is a dynamic optimisation problem. Additionally, the total proteome fractions of membrane‐bound and cytoplasmic enzymes were set based on established approaches (Jin et al. 2022).

DIET between ANME and SRB in shell‐shaped aggregates introduces two additional constraints. First, ANME and SRB must have equal methane and sulphate uptake fluxes. For aggregates with a diameter of 4 μm (Knittel and Boetius 2009), the biomass‐specific uptake flux ratio of methane to sulphate is 1.0 (see Supporting Information S4).

Second, ANME and SRB must maintain proportional ATP production fluxes to sustain their aggregate structural integrity (Orcutt and Meile 2008). Considering that ANME and SRB share similar rates of biomass synthesis (McGlynn et al. 2015; He et al. 2021), we assume an ATP production flux ratio of 1.0 between ANME and SRB. Given that ATP flux depends on substrate uptake and ATP yield, the ATP yield ratio between ANME and SRB is also 1.0.

### 2.3 Model Implementation and Analysis

The model was implemented using the software COPASI (version 4.44; build 295) (Bergmann et al. 2017). COPASI expresses concentrations in mol⋅L^−1^, which are approximately equivalent to mol⋅kg^−1^ (molal or M) under the assumption that 1 L of water is equivalent to 1 kg of water.

To solve the dual‐objective dynamic optimisation problem, we decomposed it into two single‐objective problems, maximising the ATP production fluxes of ANME and SRB separately. A Pareto front was constructed by varying the fraction (*f*_G_) of available energy allocated to ANME.

For each *f*_G_, we first maximised the ATP production fluxes of ANME and SRB independently. For the partner whose ATP production flux exceeded that of the other, we re‐computed its optimisation problem by fixing its substrate uptake flux to match the flux of the other partner. The optimal fraction *f*_G_ was identified where both partners achieve equal substrate uptake and ATP production fluxes.

We used control vector parameterisation to solve the dynamic optimisation problem by splitting it into an outer optimisation problem and an inner IVP (Villaverde et al. 2016). The outer optimisation problem searches for optimal proteome fractions of enzymes in ANME (or SRB) with the Nelder–Mead method. The inner IVP simulates the dynamics of reverse methanogenesis (or SR). The algorithm configuration, including iteration limits and numerical tolerances, follows the methodology deployed previously (Jin et al. 2022).

The simulation results include temporal profiles of metabolite concentrations, capturing the progression of the metabolic system from its initial state to steady state. These profiles illustrate how intracellular metabolite levels evolve until thermodynamic and kinetic balances are achieved. In this study, the model was applied to investigate the energy metabolism of ANME–SRB consortia in the environment with fixed methane, sulphate, bicarbonate and sulphide concentrations. Accordingly, we focus our analysis on the steady‐state simulation outcomes, which reflect the long‐term energetic behaviour of the system under stable substrate conditions.

We analysed the two submodels with metabolic control analysis (MCA) (Fell 1997). In particular, we calculated the flux control coefficients (FCCs) according to the following equation:

(4) $$\mathrm{FCC}=\frac{\phi_{i}}{J}\cdot \frac{\partial J}{\partial \phi_{i}}$$

where *ϕ*_i_ is the mass fraction of enzyme i in the proteome, and *J* is the flux.

We calculated the thermodynamic potential factor *F*_T_ according to the following equation:

(5) $$F_{T}=1-\exp\left(\frac{f}{\mathrm{\chi RT}}\right)$$

where *f* is the thermodynamic drive (J⋅mol^−1^), *χ* is the average stoichiometric number (Jin and Bethke 2007).

### 2.4 Data Availability

All model symbols, parameters and units used throughout the manuscript are defined in Table 2 for ease of reference. The kinetic model in SBML and COPASI formats is available on GitHub (https://github.com/geomicrobiology/AOM‐SR).

**TABLE 2:** Summary of model parameters, symbols and units.

Parameter	Symbol	Unit	Description
Average stoichiometric number	*χ*	dimensionless	Average number of times a reaction occurs per turnover of an overall metabolic pathway.
	*χ*_E_	dimensionless	Average number of times a rate‐limiting step occurs per turnover of an enzyme reaction.
ATP production flux	*J*_RM,ATP_; *J*_SR,ATP_	mmol·g^−1^·d^−1^	ATP production fluxes of ANME (RM) or SRB (SR) in mmol per g cell dry weight per day.
ATP yield	*Y*_ATP_	mol·mol^−1^	Mol ATP synthesised per mol of substrate.
Energy partition fraction	*f*_G_	dimensionless	Fraction of available energy allocated to ANME.
Flux control coefficient	FCC	dimensionless	Proportional change in flux in response to a proportional change in enzyme abundance.
Gas constant	*R*	J⋅mol^−1^⋅Κ^−1^	Universal gas constant.
Gibbs free energy change	Δ*G* ^o′^	kJ⋅mol^−1^	Standard Gibbs free energy change (e.g., at pH 7.0, 25°C, 1 atm, and 1M reactants and products)
	Δ*G*	kJ⋅mol^−1^	Actual Gibbs free energy change.
Membrane potential	Δ*ψ*	mV	Electrical voltage difference across the membrane.
Proteome fraction	*ϕ*_RM,i_; *ϕ*_SR,i_	%	Proteome fractions of enzyme *i* in ANME (RM) or SRB (SR).
	*ϕ*_RM,iMax_; *ϕ*_SR,iMax_	%	Maximum proteome fractions of enzyme *i* in ANME or SRB.
Reduction potential	*E*_A_, *E*_D_	mV	Reduction potential of electron acceptors and donors.
Return on investment	ROI	%	Ratio of the energy conserved as ATP to the energy invested in activating the substrate.
Stoichiometric coefficient	*ν*_RM,i+_; *ν*_SR,i+_	dimensionless	Number of ions translocated across the membrane per reaction by enzyme *i*.
	*ν*_+,max_	dimensionless	Maximum number of ions translocated across the membrane per reaction.
Thermodynamic drive	*f*	kJ⋅mol^−1^	Free energy available to drive a reaction.
Temperature	*T*	*K*	Absolute temperature.
Thermodynamic efficiency	*η*	%	The fraction of the total energy available from a redox reaction that is conserved in the form of ATP synthesis.
Thermodynamic potential factor	*F*_T_	dimensionless	The extent to which thermodynamic drive limits the rate of a reaction.

Detailed descriptions of model components, including the ODEs and initial concentrations of metabolites, the kinetic expressions of metabolic reactions, and respective thermodynamic and kinetic parameters, are provided in Supporting Information S1. These resources ensure full transparency and reproducibility of our computational analyses and provide a comprehensive framework for future investigations into AOM–SR bioenergetics.

## 3 Results

This study developed a thermodynamically constrained kinetic metabolic model to simulate the catabolic pathways of sulphate‐dependent AOM consortia. By applying the model to a typical laboratory bioreactor, we addressed three core questions regarding methane activation, energy conservation and the extent of thermodynamic limitations.

### 3.1 Comparative Analysis With Experimental Observations

We calibrated the metabolic model to reflect the physiological state of AOM consortia under typical laboratory bioreactor conditions. Previous studies have applied a wide range of methane and sulphate concentrations, often differing by orders of magnitude (see Supporting Information S5). To represent a standardised experimental setup, we assumed pH 7, 10 mM methane, 10 mM bicarbonate, 10 mM sulphate and 1 mM sulphide as the representative experimental conditions. The calibrated model was validated by comparing its outputs against independent experimental observations.

The model calibration yielded enzyme abundances, reported as proteome fractions (Table 3). In ANME, MCR was the most abundant enzyme (12.0% of the proteome), aligning with experimental estimates (7%–10%) (Krüger et al. 2003; Scheller et al. 2010). In SRB, sulphate adenylyltransferase (SAT) was the most abundant cytoplasmic enzyme (7.0%), while adenosine‐5′‐phosphosulfate reductase (APR) was the most abundant membrane‐bound enzyme (3.4%). These predictions match previous studies where SAT accounted for ~8% of the SRB proteome in microbial mats from methane seeps in the Black Sea (Basen et al. 2011) and for 8.1% in axenic SRB, with APR at 4.0% (Dahl 1990; Gavel et al. 1998).

**TABLE 3:** Enzyme properties and pathway metrics in AOM Consortia.

Microbe	Enzyme^a^	Reaction^b^	Δ*G* (kJ·mol^−1^)	*ϕ* (%)	*ν*_+_ ^c^	*χ*_E_	*f* (kJ·mol^−1^)	FCC
ANME								
	MCR (c)	CH_4_ + CoB‐S‐S‐CoM = HS‐CoB +CH_3_‐S‐CoM	−1.44	12.0	—	1	1.44	0.44
	FPO (m)	F420H_2_ + MP = F420 + MPH_2_	−27.8	4.0	1.35	2	0.87	0.36
	MTR (m)	H_4_SPT + CH_3_‐S‐CoM = CH_3_‐SPT + HS‐CoM	39.4	2.9	−2.00	1	0.46	0.08
	MER (c)	CH_3_‐H_4_SPT + F420 = CH_2_ = H_4_SPT + F420H_2_	−0.3	1.9	—	1	0.26	0.05
	FMD (c)	CHO‐MF + Fd_ox_ = MF + Fd_red_ + ${\mathrm{HCO}}_{3}^{-}$	−0.2	4.7	—	1	0.17	0.03
	FTR (c)	CHO‐H_4_SPT + MF = CHO‐MF + H_4_SPT	−6.6 × 10^−2^	0.2	—	1	0.07	0.01
	HDR (m)	HS‐CoB + HS‐CoM + MP = CoB‐S‐S‐CoM + MPH_2_	39.8	1.8	−2.00	1	0.06	0.01
	RNF (m)	2 Fd_red_ + MP = 2 Fd_ox_ + MPH_2_	−39.9	0.9	2.00	1	0.05	0.01
	MTD (c)	CH_2_ = H4SPT + F420 = CH ≡ H4SPT + F420H_2_	−4.2 × 10^−2^	0.1	—	1	0.04	0.01
	MCH (c)	CH ≡ H4SPT = CHO‐H_4_SPT	−3.0 × 10^−3^	0.9	—	1	2.95 × 10^−3^	0.00
	MRP (m)	Na^+^ + H^+^(m) = Na^+^(m) + H^+^	0.00	0.2	—	0	0.00	0.00
	ATPase (m)	ADP + P_i_ = ATP	48.9	0.1	−3.00	0.234	10.83	—^d^
SRB								
	APR (m)	APS + MQH_2_ = ${\mathrm{SO}}_{4}^{2-}$+AMP + MQ	−27.7	3.4	1.99	1	2.87	0.40
	PPA (m)	PPi = 2Pi	−14.7	2.8	0.96	1	2.65	0.39
	SULP (m)	${\mathrm{SO}}_{4}^{2-}\left(e\right)={\mathrm{SO}}_{4}^{2-}$	8.1	0.1	−0.67	1	0.27	0.06
	DSR (m)	${\mathrm{SO}}_{3}^{2-}$ + 3MQH_2_ = H_2_S + 3 MQ	−25.8	0.9	2.01	1	0.69	0.07
	QRC (m)	2TpIc_red_ + MQ = 2TpIc_ox_ + MQH_2_	−14.7	0.9	1.16	4	0.17	0.07
	SAT (c)	${\mathrm{SO}}_{4}^{2-}$ + ATP = Aps + PP_i_	−3.8 × 10^−2^	7.0	—	1	3.79 × 10^−3^	0.01
	ADK (c)	AMP + ATP = 2 ADP	−1.7 × 10^−3^	3.0	—	1	1.66 × 10^−3^	0.00
	ATPase (m)	ADP + P_i_ = ATP	48.9	1.9	−4.00	2.235	0.94	—^d^

*Note:* This table summarises key properties of enzymes involved in reverse methanogenesis and dissimilatory sulphate reduction in AOM consortia under typical laboratory bioreactor conditions. Listed parameters include reaction equations, Gibbs free energy changes (Δ*G*), enzyme proteome fractions (*ϕ*), ion translocation stoichiometries *ν*_+_, stoichiometric numbers *χ*_E_ of times enzyme reactions take place per methane molecule or sulphate anion, thermodynamic drives *f* of enzyme reactions and their flux control coefficients (FCCs).

^a^ (m) denotes membrane‐bound enzymes and metabolites, and (c) indicates cytoplasmic enzymes.

^b^ Only enzyme‐catalysed reactions are listed in this table. For details on diffusion processes transporting methane, sulphide and dissolved carbon dioxide across the membranes, please refer to Supporting Information S1; metabolites labelled with (e) denote extracellular compounds, while unlabelled metabolites (e.g., CH_4_, H_2_S and others) refer to species located within the cytoplasm or associated with the membrane.

^c^ Negative values indicate ion translocation inward across the membrane.

^d^ ATP synthases are excluded from the metabolic control analysis because in the model, ATP, ADP and Pi concentrations are fixed, and ATP synthesis is at steady state.

The model predicts that ANME and SRB generate membrane electrical potentials of 206 and 129 mV, respectively (Table 1). Axenic SRB are known to produce membrane potentials ranging from 85 to 155 mV (Jin 2012), supporting the plausibility of these predictions.

The simulated metabolic fluxes indicate that ANME oxidise methane and SRB reduce sulphate at an equal rate of 20 mmol·g^−1^·d^−1^. Similarly, enrichments of AOM consortia show specific SR rates of up to 20 mmol·g^−1^·d^−1^ (Knittel and Boetius 2009).

These results demonstrate the model's agreement with experimental data and confirm its utility for investigating the energy metabolism of ANME–SRB consortia.

### 3.2 MCA

To evaluate the robustness of the metabolic model and identify key regulatory enzymes, we performed a sensitivity analysis by computing FCCs (Fell 1997; Plaxton 2004). FCC values near 1 indicate that an enzyme exerts significant control over pathway fluxes, whereas values close to 0 indicate negligible control. Furthermore, enzymes with markedly higher FCC values than others in the same pathway are classified as key regulatory or rate‐limiting.

In the reverse methanogenesis pathway, control of methane uptake flux is unevenly distributed across the enzymes, indicating potential rate‐limiting steps (Table 3). MCR has the highest FCC value of 0.51, indicating strong control over methane uptake. Similarly, F420H_2_ oxidoreductase (FPO) has an FCC of 0.33 and therefore also exerts substantial control. In contrast, the remaining enzymes have FCC values < 0.1, suggesting secondary control. These results identify MCR and FPO as the two primary rate‐limiting enzymes in reverse methanogenesis.

In the dissimilatory SR pathway, control of sulphate uptake flux is also unevenly distributed. APR and pyrophosphatase (PPA) have the highest FCCs, ~0.3, indicating their critical roles in regulating pathway fluxes. The remaining enzymes have FCC values < 0.1. These results suggest that APR and PPA are two rate‐limiting enzymes in SR.

The computed FCC values align with prior studies, further supporting the reliability of our metabolic model. Previous studies have identified MCR as a rate‐limiting enzyme in ANME, consistent with our FCC value of 0.51 (He et al. 2022; Scheller et al. 2010). Similarly, the FCC values for APR and PPA (~0.3) corroborate their well‐documented rate‐limiting effects in SR (Santos et al. 2015; Leavitt et al. 2015; Leyh 1993). These results also align with metabolic control theory (Fell 1997; Plaxton 2004), underscoring the distributed nature of metabolic control.

### 3.3 Overcoming Thermodynamic Barriers

Both methane and sulphate are chemically inert and require substantial energy investment for activation before oxidation or reduction (Leyh 1993; Thauer and Shima 2008). Here, we analyse simulation results to demonstrate how ANME overcome the thermodynamic barrier of methane activation and how SRB facilitate sulphate activation through the enzymatic pathways in Figure 1.

#### 3.3.1 Methane Activation

ANME utilise MCR to activate methane to methyl‐coenzyme M (CH_3_‐SCoM)—a reaction that operates in reverse of its role in methanogenesis (Krüger et al. 2003; Scheller et al. 2010). The reverse MCR reaction is thermodynamically challenging, with a standard Gibbs free energy change (Δ*G* ^o′^) of +17 kJ per mol of dissolved CH_4_ (Thauer 2019; Tietze et al. 2003).

Steady‐state simulations reveal that specific metabolite concentrations can drive the reverse MCR reaction towards thermodynamic favourability. For instance, when heterodisulfide (CoMS‐SCoB) accumulates to 1.6 mM, while CH_3_‐SCoM and coenzyme B (HSCoB) remain at 0.36 mM and 31.1 μM, respectively, the resulting concentration profile shifts the Gibbs free energy change (Δ*G*) of the reverse MCR reaction to −1.4 kJ⋅mol^−1^, rendering it thermodynamically favourable. Therefore, maintaining low CH_3_‐SCoM and HSCoB concentrations, alongside high CoMS‐SCoB levels, sustains the thermodynamic favourability of the reverse MCR reaction.

#### 3.3.2 Enzyme Roles in Metabolite Regulation

**FIGURE 2:**
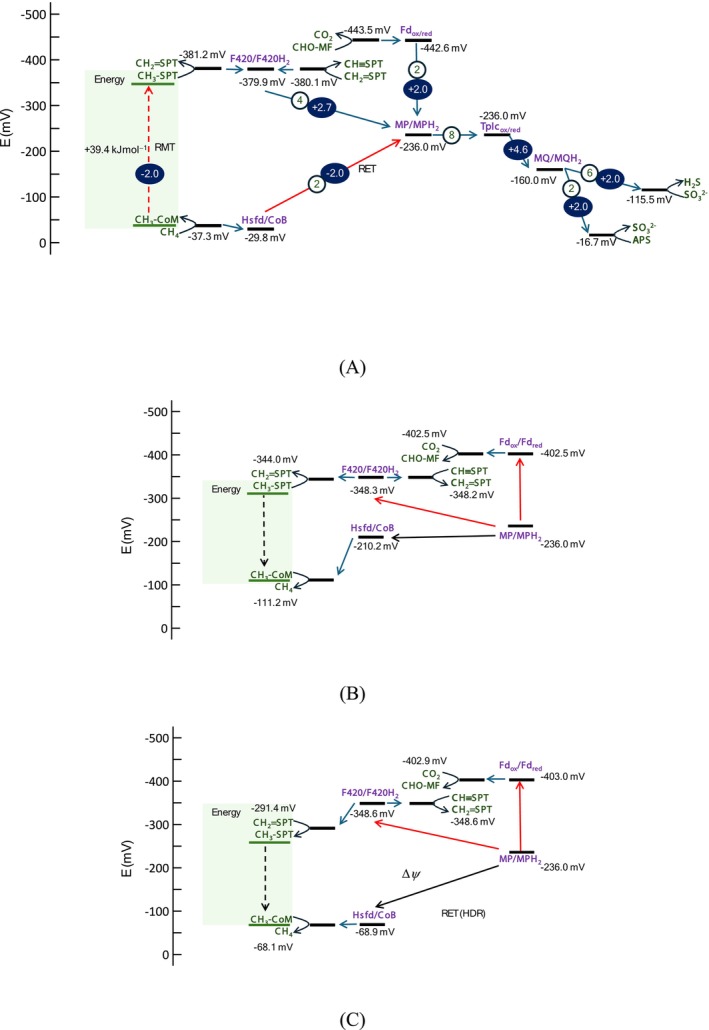
Diagram illustrating the redox potentials of C_1_‐carrying compounds (dark green) in reverse methanogenesis, redox‐active metabolites (purple), and intermediate compounds of sulphate reduction where both RET and RMT are active (A), RET is not active (B) or RMT is not active (C). Numbers in open circles are the number of electrons transferred, and the numbers in dark ovals are the number of ions translocated per methane molecule or sulphate anion. Solid black arrows represent exergonic electron transfer, while red arrows depict reverse electron transfer (RET) catalysed by HDR. The red dashed arrow in the shaded area indicates the reverse methyl‐group transfer (RMT) catalysed by MTR. The redox potential values of metabolites are indicated by black bars, while green bars in the shaded area illustrate the relative energy levels of CH_3_‐SPT and CH_3_‐CoM in methyl‐group transfer. The Gibbs free energy change of endergonic methyl‐group transfer is shown within the shaded area. Full metabolite names are available in the caption of Figure 1.

Heterodisulfide reductase (HDR) plays a central role in maintaining high CoMS‐SCoB and low HSCoB concentrations (Figure 2A). HDR oxidises HSCoB and coenzyme M (HSCoM) to produce CoMS‐SCoB and transfers the released electrons to the reduction of methanophenazine. Concurrently, HDR also translocates one proton per electron inward across the membrane. This electron transfer is thermodynamically uphill, as the reduction potential of HSCoB and HSCoM is 206 mV higher than that of methanophenazine. However, the proton translocation dissipates the membrane potential, providing the necessary energy to drive the otherwise endergonic electron transfer—a process known as reverse electron transfer (RET) (Sieber et al. 2018). By using RET, ANME not only transfer electrons uphill against the redox potential gradient but also hold HSCoB concentration low and CoMS‐SCoB concentration high.

The low CH_3_‐SCoM concentration is maintained by methyltransferase (MTR). MTR transfers the methyl group from CH_3_‐SCoM to tetrahydromethanopterin (H_4_SPT), a reaction with a Gibbs free energy change of +39.4 kJ⋅mol^−1^. To overcome this barrier, MTR translocates two sodium cations inward per methyl group transferred. The sodium cation translocation dissipates the membrane potential, which not only keeps the MTR reaction thermodynamically favourable but also maintains CH_3_‐SCoM concentrations low. Analogous to RET, this coupling mechanism can be termed reverse methyl transfer (RMT) to emphasise the role of the membrane potential in driving methyl group transfer.

Both RET and RMT are essential for methane activation. To illustrate their importance, we conducted a simulation assuming that HDR catalyses electron transfer without proton translocation. Under these conditions, the model predicted that ANME would hydrolyse ATP to reduce CO_2_ to CH_4_, an energetically unsustainable scenario (Figure 2B). Likewise, when assuming that MTR transfers the methyl group without translocating the sodium cation, the model without RMT also predicted ATP hydrolysis to produce CH_4_ (Figure 2C).

#### 3.3.3 Sulphate Activation

SRB activate sulphate through ATP sulfurylase (SAT), which combines sulphate with ATP to form adenosine‐5′‐phosphosulfate (APS) and pyrophosphate (PP_i_). This reaction has a standard Gibbs free energy change (Δ*G* ^o^′) of +46.4 kJ⋅mol^−1^ (Leyh 1993). However, steady‐state simulations show that cytoplasmic concentrations of sulphate (262.5 mM), ATP (10 mM), PP_i_ (5.9 μM) and APS (3.3 μM) shift the Gibbs free energy change to −37.9 J⋅mol^−1^, allowing the reaction to proceed spontaneously in situ.

This thermodynamic reversal results from the coordinated actions of enzymes PPA and APR (Leyh 1993). PPA maintains low PP_i_ concentrations via hydrolysis. APR keeps APS concentrations low by reducing APS to sulphite. The combined actions of SAT, PPA and APR make sulphate activation energetically equivalent to the consumption of two moles of ATP per mol sulphate.

The thermodynamic reversal is further facilitated by the sulphate/proton symporter (SULP). SULP uses the membrane potential to import sulphate into the cytoplasm, translocating 0.67 protons per sulphate anion. This process generates a cytoplasmic sulphate concentration 26 times higher than the external environment, creating a steep concentration gradient that supports the activation process.

Taken together, the energetic cost of sulphate activation—including ATP consumption by SAT and the proton motive force associated with SULP‐mediated transport—is approximately 2.2 ATP equivalents per sulphate anion.

### 3.4 Energy Conservation Mechanisms and Evaluation

We next applied the metabolic model to investigate the mechanisms of energy conservation in ANME and SRB and to evaluate their conservation efficiencies. Given the low energy yields of AOM‐SR reactions [4], these energy conservation mechanisms and efficiencies are fundamental to their ability to thrive under thermodynamic constraints.

#### 3.4.1 Energy Partition

In the simulated bioreactor, the AOM‐SR reaction releases 38.6 kJ⋅(mol CH_4_)^−1^ (Table 1 and Figure 2), which is partitioned nearly equally between ANME and SRB. DIET between the two organisms is mediated via type I tetrahaem cytochrome c3 (TpIc3), which maintains a steady‐state reduction potential of −236.0 mV. Consequently, ANME receive 20.4 kJ⋅(mol CH_4_)^−1^ and SRB receive 18.2 kJ⋅(mol SO_4_^2−^)^−1^.

#### 3.4.2 Chemiosmotic Energy Conservation

Both ANME and SRB conserve energy by translocating protons and sodium cations outward across the membrane. Metagenomic and metatranscriptomic studies have linked ANME's energy conservation to FPO and the *Rhodobacter* nitrogen fixation complex (RNF) (Wang et al. 2014; Hallam et al. 1979). Simulation results indicate that these enzymes contribute differently to energy conservation: RNF translocates one sodium cation, while FPO translocates 0.68 protons per electron (Table 3). Considering the inward ion translocation via RET and RMT, a net 0.70 charge is translocated out of the membrane per methane molecule.

The dissimilatory SR pathway utilises three ion‐translocating redox enzymes: APR, quinone‐reductase complex (QRC) and dissimilatory sulphite reductase (DSR) (Pereira et al. 2011). Simulations indicate that these enzymes translocate 1.0, 0.58 and 0.34 protons per electron transfer, respectively. Additionally, the PPA enzyme translocates one proton per pyrophosphate molecule. Considering the inward ion translocation by SULP, a net charge of 8.94 is translocated out of the membrane per sulphate anion.

**FIGURE 3:**
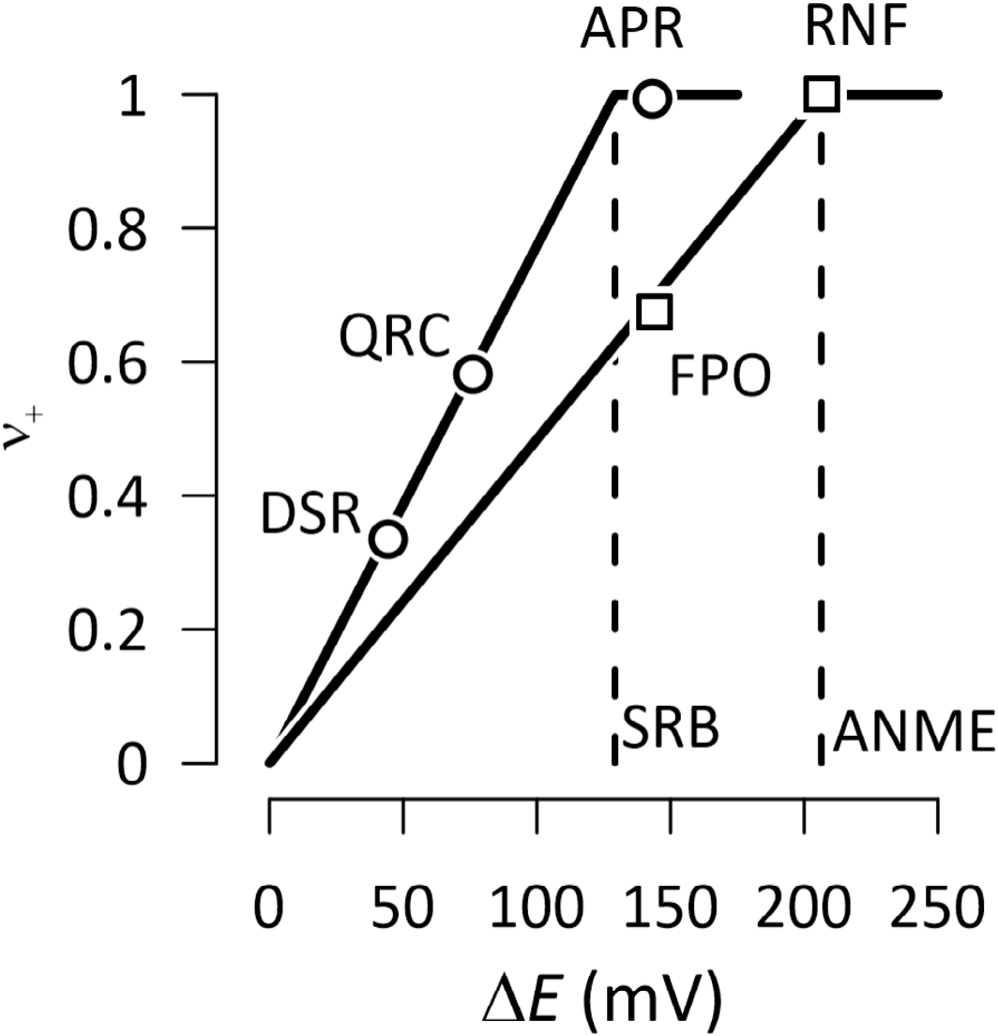
Variations with the redox gradient (Δ*E*) in the number (*ν*_+_) of protons or sodium cations translocated from the inside to the outside of the membrane per electron transfer by the enzymes of ANME (□) and SRB (○). Data points represent simulation results, while solid lines indicate predicted ion translocation numbers based on the ratio of redox gradient (Δ*E*) to membrane potential, with a maximum stoichiometry of 1 ion per electron. Vertical dashed lines denote the simulated membrane potentials for ANME (206 mV) and SRB (129 mV). Enzyme abbreviations are provided, with full names available in the caption of Figure 1.

In both ANME and SRB, ion translocation stoichiometries are influenced by redox potential gradients relative to the membrane potential (Figure 3). Where the redox gradients exceed the membrane potentials, as in RNF and APR, the stoichiometry reaches its maximum of one ion per electron. In contrast, for FPO, QRC and DSR, where the redox gradient is lower than the membrane potential, stoichiometries scale proportionally with the redox gradient. These findings explain the varying contributions of ion‐translocating enzymes to energy conservation (see Table 3). They suggest that the ion translocation stoichiometries are not fixed, but are fine‐tuned to align with the redox chemistry of catabolic pathways.

#### 3.4.3 ATP Synthesis

ATP synthesis is the primary driver of syntrophic interactions between ANME and SRB. We quantify energy conservation with three metrics: ATP yield, thermodynamic efficiency (Decker et al. 1970) and ROI. ROI, the ratio of the energy conserved through ATP synthesis to the energy invested in substrate activation, captures the trade‐off between energy gain and expenditure. These metrics offer complementary perspectives on energy metabolism.

The stoichiometry of proton translocation by ATP synthase varies depending on the membrane potential (Fischer et al. 2000; Cross and Müller 2004). In ANME, with a membrane potential of 206 mV, three protons are assumed to be translocated per ATP synthesised. In contrast, SRBs, which operate with a lower membrane potential of 129 mV, require four protons per ATP.

Based on these values, ANME yield 0.234 mol ATP per mol of methane, while SRB make 0.235 mol ATP per mol of sulphate (Table 1). These predicted yields are on the lower end of values reported for other microbial systems, such as methanogenesis and SR (see Supporting Information S6), reflecting the significant thermodynamic constraints of the AOM‐SR reaction. Nevertheless, the predictions are consistent with experimental observations. Assuming an ATP‐to‐biomass conversion of 5 g per mol ATP (Jin 2012) and a 50% allocation of ATP to biomass synthesis (Thauer et al. 1977), both ANME and SRB are predicted to produce 0.6 g of cell dry weight per mol methane or sulphate. These estimates align with laboratory incubation studies reporting a biomass yield of 0.6 g⋅(mol CH_4_)^−1^ for ANME (Nauhaus et al. 2007).

From these yields, the thermodynamic efficiency of energy conservation is calculated as 62.7% for ANME and 56.5% for SRB, leading to an overall efficiency of 59.4%. Additionally, ANME exhibit a higher ROI of 18%, compared to 11% for SRB, highlighting differences in energetic strategy between the two partners.

### 3.5 Thermodynamic Limitation on AOM‐SR Kinetics

The low energy yields of AOM‐SR reactions are widely recognised as a limiting factor for reaction rates (Knittel and Boetius 2009; Dale et al. 2006; Wegener et al. 2021). Here we apply our model to quantify the extent to which AOM‐SR kinetics are constrained by thermodynamic drives—the difference between the energy allocated to ANME (or SRB) and the energy conserved by the organisms. We base our evaluation on the thermodynamic potential factor *F*_T_ (Equation 5), a dimensionless parameter that quantifies the extent to which the thermodynamic drive limits the rate of a reaction (Jin 2023). It ranges from 0 to 1, where an *F*_T_ value of 1 indicates no limitation (i.e., the reaction is far from equilibrium and can proceed at maximum rate), while values approaching 0 suggest reactions operating close to equilibrium, with significant kinetic limitation. *F*_T_ thus captures how available free energy (Δ*G*) translates into effective reaction fluxes in energy‐limited systems such as AOM.

#### 3.5.1 Thermodynamic Drives

Under the simulated laboratory bioreactor conditions, reverse methanogenesis has a thermodynamic drive of 4.3 kJ⋅(mol CH_4_)^−1^, and dissimilatory SR operates with a drive of 8.6 kJ⋅(mol ${\mathrm{SO}}_{4}^{2-}$)^−1^. The different thermodynamic drives stem from the distinct stoichiometries of ion translocation and the membrane potentials of ANME and SRB (see Table 1).

Thermodynamic drives are unevenly distributed among enzyme‐catalysed reactions within the reverse methanogenesis and the SR pathways (Table 3). In reverse methanogenesis, the rate‐limiting enzymes MCR and FPO consume 1.4 and 0.9 kJ·(mol CH_4_)^−1^, respectively. In SR, the rate‐limiting APR and PPA consume 2.9 and 2.7 kJ·(mol SO_4_^2−^)^−1^, respectively. In contrast, the remaining enzymes in both pathways utilise < 0.7 kJ·mol^−1^.

The uneven distribution of thermodynamic drives is characterised by the average stoichiometric number (*χ*), which reflects the frequency with which key enzyme reactions occur along a pathway (Jin and Bethke 2007). ANME possess two key enzymes with relatively large FCCs: MCR and FPO. The corresponding *χ* value is ~2.5 per methane molecule, as the MCR reaction occurs once while the FPO reaction occurs twice per methane molecule. In SRB, the key enzymes are APR and PPA, each of which occurs once per sulphate anion, resulting in a *χ* value of ~2.0 per sulphate anion.

**FIGURE 4:**
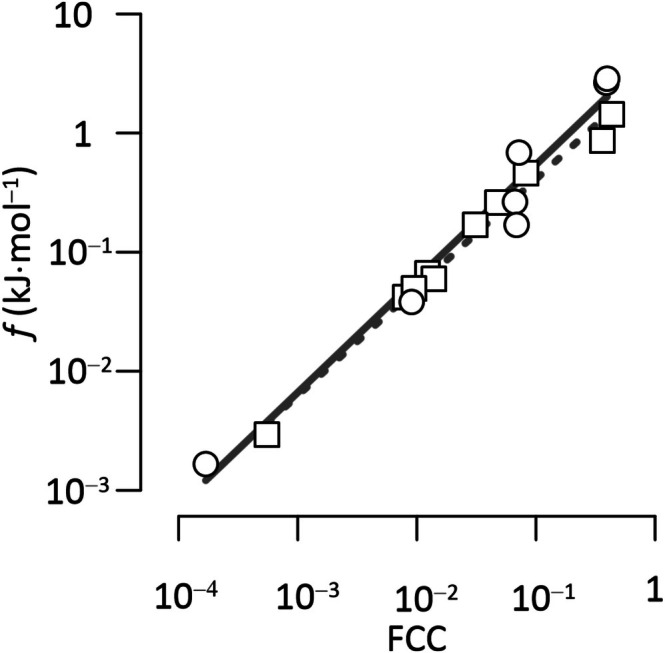
Strong correlations between thermodynamic drives *f* and flux control coefficients (FCCs) of enzymes in reverse methanogenesis (□) and dissimilatory sulphate reduction pathways (○). Solid and dashed lines are the best fit for the enzymes of sulphate reduction and reverse methanogenesis, respectively. The plot highlights that rate‐limiting enzymes with high FCC values (e.g., MCR and FPO in reverse methanogenesis; APR and PPA in sulphate reduction) consume significant thermodynamic drives, reflecting their central roles in pathway regulation.

To examine the relationship between enzyme thermodynamics and flux control, we performed correlation analyses between thermodynamic drives and FCCs of enzymes (Figure 4). The analyses revealed strong positive correlations, with Spearman correlation coefficients of 0.99 for reverse methanogenesis (*n* = 11, *p* < 0.01) and 0.96 for SR (*n* = 7, *p* < 0.01). These results indicate that key enzymes, such as MCR and FPO from ANME and APR and PPA from SRB, which consume a significant portion of the thermodynamic drives, also serve as key rate‐limiting reactions in their respective pathways.

#### 3.5.2 Kinetic Impact

Differences in thermodynamic drive and average stoichiometric number result in varying degrees of thermodynamic constraints for the two pathways (Table 1). The reverse methanogenesis pathway has an *F*_T_ value of 0.51, indicating that thermodynamic limitations reduce the net reaction rate by approximately half—reflecting strong thermodynamic control. In contrast, the SR pathway has an *F*_T_ value of 0.84, suggesting only moderate thermodynamic constraints on its reaction kinetics.

## 4 Discussion

This study provides a mechanistic framework for understanding how sulphate‐dependent AOM consortia conserve energy under extreme thermodynamic constraints. By integrating enzyme‐level thermodynamics and kinetics, the model reveals how ANME and SRB sustain metabolism at the energetic edge of viability. These insights lay the foundation for exploring the fundamental bioenergetics of syntrophic microbial interactions and their implications for methane cycling, ecosystem modelling and mitigation strategies.

### 4.1 Energy Investment in AOM‐SR Reactions

Previous studies of sulphate‐dependent AOM have debated the roles of RMT and RET, with some proposing that MTR supports ion‐coupled methyl transfer (RMT), and others positing electron transfer through HDR with or without associated proton translocation (RET) (Wang et al. 2014; McGlynn et al. 2015; McGlynn 2017; Timmers et al. 2017). Our findings clarify that both RMT and RET are essential for methane activation under sulphate‐reducing conditions. This combined RMT and RET mechanism allows ANME to overcome the high energetic barrier of methane oxidation without direct ATP consumption, by leveraging ion translocation to maintain redox balance and generate a sufficient thermodynamic driving force. Although this strategy is proteomically and energetically costly—due to the need for multiple ion‐translocating enzymes, it enables efficient energy conservation under low‐energy environmental conditions.

In contrast, SRB invest more energy upfront, particularly in sulphate transport and activation. Together, these steps require approximately 2.2 ATP equivalents per sulphate anion. While SR downstream conserves energy via electron transport phosphorylation, the initial activation cost is not directly recovered. It is a necessary investment to initiate SR. This higher energy investment contributes to the lower ROI observed in SRB (11%) compared to ANME (18%), highlighting the energetic burden imposed by substrate activation in sulphate‐dependent AOM.

### 4.2 Energy Conservation Mechanisms and Efficiencies

Our model integrates enzyme‐level thermodynamics and kinetics to dynamically simulate chemiosmotic ion translocation. Unlike previous stoichiometric metabolic models that imposed fixed ion translocation stoichiometries (Arshad et al. 2015; He et al. 2022), this mechanistic framework allows for variable, enzyme‐specific translocation, yielding a more accurate and adaptable representation of microbial energy conservation.

A key insight from our results is that ANME and SRB exhibit unusually high thermodynamic efficiencies (~60%) of ATP synthesis, especially considering the low energy yield of the AOM‐SR pathway. For comparison, most anaerobic processes operate at efficiencies between 15% and 30%, while even aerobic glucose respiration efficiencies rarely exceed 46% (Decker et al. 1970). These results suggest that both organisms have evolved highly optimised energy conservation strategies to sustain growth in energetically marginal environments.

Importantly, these high efficiencies do not result from rigid stoichiometric configurations, but from adaptive tuning of ion‐translocating enzymes in response to the redox potential gradient (Figure 3). This dynamic flexibility allows the consortia to sustain ATP production under variable environmental conditions by modulating enzyme activity according to metabolic demand.

Moreover, the model's predictions are consistent with observed biomass yields in experimental systems, further supporting the biological plausibility of these conservation strategies. Collectively, these results suggest that syntrophic consortia like ANME–SRB have evolved not merely to survive at thermodynamic limits, but to effectively exploit marginal energy resources.

### 4.3 Thermodynamic Constraints on AOM‐SR Kinetics

The inherently low energy yields of AOM‐SR reactions result in small thermodynamic drives, which are widely recognised as a fundamental limitation on reaction kinetics (Knab et al. 2008; Wegener et al. 2021). Our model supports this view by quantifying thermodynamic limitations with the thermodynamic potential factor (*F*_T_) and showing that reverse methanogenesis operates under greater thermodynamic constraint than SR, a trend consistent with environmental isotope‐labelling studies across diverse ecosystems. Calculations from these studies reveal *F*_T_ values for reverse methanogenesis are as low as 0.37 in a microbial community from the Black Sea (Seifert et al. 2006), and 0.65 in a community from the cold seeps of the Gulf of Mexico (Orcutt et al. 2008). Additionally, *F*_T_ values derived from the data of Holler et al. (2011) indicate differential thermodynamic control, with an *F*_T_ of 0.95 for methane oxidation and 0.88 for SR. These results suggest that thermodynamic limitations, rather than sulphate and methane concentrations alone, govern the achievable rates of AOM–SR reactions.

Our model also reveals that thermodynamic drives are not evenly distributed across all enzymatic steps, but are rather channelled through a small subset of rate‐limiting enzymes—notably MCR and FPO in reverse methanogenesis, and APR and PPA in SR. Such uneven energy distribution mirrors patterns observed in other metabolic pathways, such as methanogenesis from methanol (Jin et al. 2022), glycolysis (Chandel 2021) and the citric acid cycle (Miller and Smith‐Magowan 1990), where only a few key enzymes account for most of the thermodynamic drives. The strong correlation between thermodynamic drives and FCCs (Figure 4) supports the thermodynamic control theory of metabolic regulation, which suggests that energy‐limited pathways are constrained by the availability of free energy rather than enzyme abundance alone.

### 4.4 Model Limitations and Future Work

Our model was calibrated under typical laboratory conditions, where methane and sulphate concentrations are relatively high (e.g., 10 mM methane and 10 mM sulphate). These conditions result in a relatively favourable energy yield of 38.6 kJ⋅(mol CH_4_)^−1^, which may not fully reflect the thermodynamic conditions typically encountered by these consortia in nature (Knittel and Boetius 2009). Notably, these substrate levels approach or exceed the reported half‐saturation constants of ANME‐SRB consortia (e.g., ~10 mM for methane and < 1 mM for sulphate; Knittel and Boetius 2009; Wegener and Boetius 2009).

Our results also suggest that both ANME and SRB dynamically adjust their ion‐translocation stoichiometries to optimise energy conservation. Under more substrate‐ and energy‐limited conditions, different values for the methane consumption flux, ATP yield, thermodynamic efficiency and ROI would likely emerge. Future simulations should examine the metabolic adaptations of AOM consortia under such limiting conditions, which are more representative of deep marine sediments and other energy‐poor environments.

Moreover, while this study focuses on canonical sulphate‐dependent AOM, we acknowledge that ANME are metabolically versatile and can form syntrophic associations with partners utilising alternative electron acceptors, such as nitrate, nitrite, Mn(IV), Fe(III) oxyhydroxides and humic substances (Knittel and Boetius 2009). ANME may also reduce sulphate to elemental sulphur (S^0^), which is then disproportionated by SRB (Milucka et al. 2012). These alternative metabolic pathways may operate under specific environmental conditions, but remain poorly understood.

We selected the canonical SR pathway for this study due to its strong experimental support and the availability of mechanistic parameters suitable for enzyme‐level modelling. Nevertheless, our framework is modular and extensible. With ongoing advances in metagenomics, proteomics and biochemical characterisation, this model can be adapted to incorporate alternative AOM pathways and intermediate sulphur species (e.g., S^0^, polysulfides and extracellular electron shuttles). Such developments would enable a deeper and more comprehensive understanding of AOM metabolism across a wide range of anoxic ecosystems.

### 4.5 Broader Implications and Integrated Perspectives

Our findings provide key insights into the bioenergetics of sulphate‐dependent AOM consortia, with broad implications for biogeochemistry, methane mitigation and biotechnological applications. By characterising energy conservation strategies in detail, our study reveals how AOM consortia sustain metabolism on the thermodynamic edge—where energy yields are minimal and metabolic balance is critical. These findings enhance our understanding of how microbial energy metabolism shapes methane fluxes, offering a framework for integrating enzyme‐level thermodynamics and kinetics into ecosystem‐scale models. Given the critical role of AOM in regulating global methane emissions (Reeburgh 2007), incorporating these constraints into predictive models will improve methane flux estimates in response to climate change and environmental perturbations.

Beyond biogeochemical modelling, our results provide strategies for engineering methane removal systems. The near‐equal partitioning of energy between ANME and SRB suggests that maintaining thermodynamic balance is essential for syntrophic stability. This insight can inform bioreactor design, where optimising substrate availability (e.g., methane and sulphate) and removing inhibitory products (e.g., bicarbonate and sulphide) could enhance methane oxidation rates. Additionally, supplementing alternative electron acceptors (e.g., nitrate and Fe^3+^) may reduce metabolic energy demand and accelerate methane removal in engineered systems (Arshad et al. 2015; He et al. 2022; Yan et al. 2018).

The proportional relationship between ion translocation and redox gradients suggests that modulating ion translocation stoichiometries or adjusting redox potential gradients could enhance microbial energy conservation, thereby improving methane oxidation efficiency (Gao et al. 2022). Identifying MCR, FPO, APR and PPA as key metabolic control points further provides potential targets for metabolic engineering to improve methane oxidation performance in both natural and industrial settings. These insights also have implications for the design of synthetic microbial consortia aimed at methane bioconversion and carbon sequestration.

At a fundamental level, our findings emphasise that syntrophic microbial interactions are inherently constrained by energy availability and conservation efficiency. In natural environments, methane fluxes are shaped not only by the presence of genes or pathway composition but also by the energetic constraints governing microbial energy conservation and ATP synthesis (Dale et al. 2006; Knab et al. 2008). These principles likely extend to other thermodynamically constrained microbial processes, such as methanogenesis, SR and syntrophic organic carbon degradation. Additionally, the near‐equal partitioning of energy between ANME and SRB reinforces the importance of metabolic interdependence in syntrophic communities, highlighting a fundamental constraint on cooperative microbial interactions.

Looking ahead, experimental validation combined with metabolic modelling will be essential to refine these predictions and broaden their application to both natural and engineered systems. As global methane emissions continue to rise, understanding and leveraging microbial methane oxidation remain critical for developing sustainable climate mitigation strategies. By bridging the gap among enzyme reactions, microbial physiology and ecosystem‐scale processes, this study provides a foundation for future research into the bioenergetics of methane cycling and its role in global carbon regulation.

## Author Contributions

Q.J. conceptualized and designed the project. Z.J. developed the initial model framework. G.B. finalized the model and conducted the result analysis. All authors contributed to the writing and revision of the manuscript.

## Conflicts of Interest

The authors declare no conflicts of interest.

## Supporting information


**Data S1.** Supporting Information.

**Data S2.** Supporting Information.

## Data Availability

The data that support the findings of this study are openly available in GitHub at https://github.com/BioGeoGordon/GroundwaterRedoxPotential.
