# Supplementary material for: Exploring Energy Conservation in Sulphate‐Dependent Anaerobic Methane‐Oxidising Consortia Through Metabolic Modelling
Source: Environ Microbiol. 2025 Jul 24;27(7):e70156. doi: 10.1111/1462-2920.70156 (PMC12287890; doi:10.1111/1462-2920.70156)
Supplement: Supplementary file 1 — Data S1. Supporting Information. [file EMI-27-e70156-s002.pdf]

Exploring Energy Conservation in Sulfate-Dependent Anaerobic Methane-Oxidising Consortia through Metabolic Modeling

Gordon Bowman, Zena Jensvold, and Qusheng Jin

Geobiology Group, University of Oregon, Eugene, OR 97405, USA.

Supplementary Material

S1. Supplementary Dataset 1 1

S2. Justification for Using Methanogen Enzyme Kinetics 1

S3. Diffusive Flux Modeling 2

S4. Flux Constraints by Syntrophic Interactions 3

S5. Chemical conditions in laboratory bioreactors 4

S6. ATP Yields 5

S7. References 5

S1. Supplementary Dataset 1

Dataset 1 (Dataset_1.xlsx) contains model formulation and parameter values used for model calibration and validation. It includes:

1. **Metabolite fluxes and differential equations.** Ordinary differential equations that compute metabolite production (or consumption) fluxes based on stoichiometric combinations of enzyme reaction rates and diffusive fluxes.
2. **Metabolic reactions and kinetics.** Stoichiometric reaction equations and corresponding reaction rate equations formulated with the reversible Michaelis-Menten equation.
3. **Enzyme kinetic and thermodynamic parameters**. Kinetic constants, Michaelis constants, and equilibrium constants for enzymes involved in reverse methanogenesis and sulfate reduction.

This dataset provides the necessary inputs for reproducing our computational analyses and serves as a resource for researchers interested in AOM-SR bioenergetics.

S2. Justification for Using Methanogen Enzyme Kinetics

Enzyme kinetic parameters for sulfate-reducing bacteria (SRB) within AOM consortia were sourced from related axenic sulfate-reducing species, ensuring consistency with experimentally derived values. However, for anaerobic methanotrophic archaea (ANME), direct biochemical characterization remains unavailable due to the lack of pure culture isolates. As a result, enzyme kinetic parameters for ANME were inferred from their closest functional and evolutionary relatives—methanogenic archaea.

This approach is supported by Kojima et al. (2014) [1], who expressed key enzymes (FTR, MCH, and MTD) from the ANME-1 metagenome in *Escherichia coli* and demonstrated that their catalytic properties closely resemble those of methanogens. Their findings indicate that despite the metabolic divergence of ANME, the core enzyme kinetics remain comparable to those of methanogenic archaea, justifying their use as surrogate models.

To further validate this approach, we examined the methyl-coenzyme M reductase (MCR), a key enzyme in both methanogenesis and reverse methanogenesis. In *Methanothermobacter marburgensis*, MCR catalyzes methane formation from methyl-coenzyme M with a maximum specific activity ($V_{\mathrm{max},+}$) of 30 U·mg^{-1} (or µmol·s^{-1}·mg^{-1}) [2]. Given the equilibrium constant (K_{eq}) of the reaction, the specific activity ($V_{\mathrm{max},-}$) for the reverse reaction (methane oxidation to methyl-coenzyme M) can be related to $V_{\mathrm{max},+}$ according to

$$K_{\mathrm{eq}} = \left(\frac{V_{\mathrm{max},+}}{V_{\mathrm{max},-}} \right)^{\chi_{\mathrm{rd}}} \tag{1}$$

where χ_{rd} is the number of times the rate-limiting step occurs per reaction, assumed to be 2 per methane molecule [3]. Based on this formulation, we estimate the $V_{\mathrm{max},-}$ value at 0.07 U·mg^{-1}. The Michaelis constant for methane is >2 mM. In laboratory experiments where methane concentration is 1 mM [2], MCR catalyzing methane oxidation would have activities <0.02 U·mg^{-1}, aligning with experimental measurements (0.01 U·mg^{-1}).

These findings collectively support the use of methanogen-derived kinetic parameters as reasonable surrogates for ANME enzymes, ensuring that our metabolic model remains biochemically grounded while acknowledging the current limitations in direct ANME enzyme characterization.

S3. Diffusive Flux Modeling

Diffusive flux between the environment and ANME-SRB aggregates is modeled using Fick's law, following the approach described by Fiksen et al. [4],

$$J_{\mathrm{j}} = 4\pi D_{\mathrm{j}} \frac{r}{n_{\mathrm{cell}}} \left(C_{\mathrm{j,env}} - C_{\mathrm{j,s}} \right), \tag{2}$$

where D_{j} is the diffusion coefficient (m^2·s^{-1}), r is the aggregate radius, n_{cell} is the number of cells on or near aggregate surface (see table S1), and $C_{\mathrm{j,env}}$ and $C_{\mathrm{j,cyto}}$ are the concentrations in the environment and cytoplasm, respectively.

This formulation integrates aggregate geometry, cell density, and concentration gradients to provide a quantitative framework for simulating metabolite exchange between microbial consortia and their surrounding environment. By incorporating these factors, the model captures the spatial constraints and diffusion-limited interactions that regulate metabolic fluxes in ANME-SRB aggregates.

S4. Flux Constraints by Syntrophic Interactions

Sulfate-dependent AOM consortia exhibit diverse morphologies, including the shell-shaped aggregates, where an inner core of ANME cells is surrounded by an outer shell of SRB cells. The average diameter of these consortia is 3 to 5 μm [5].

Sulfate-dependent AOM consortia oxidize methane by reducing sulfate reduction. By neglecting the methane and sulfate consumption by anabolic reactions [6], the uptake fluxes of methane and sulfate must be balanced

$$W_{\mathrm{ANME}} J_{\mathrm{CH_4}} = W_{\mathrm{SRB}} J_{\mathrm{SO_4^{2-}}}, \tag{3}$$

where $J_{\mathrm{CH_4}}$ and $J_{\mathrm{SO_4^{2-}}}$ represent the uptake fluxes expressed in cell dry weight, and W_{ANME} and W_{SRB} denote the dry weight of ANME and SRB cells within the consortia. Since cell dry weight is proportional to cell number n and dry weight per cell, methane and sulfate uptake fluxes per unit dry weight can be expressed as

$$\frac{J_{\mathrm{CH_4}}}{J_{\mathrm{SO_4^{2-}}}} = \frac{n_{\mathrm{SRB}} \cdot V_{\mathrm{SRB}}}{n_{\mathrm{ANME}} \cdot V_{\mathrm{ANME}}}, \tag{4}$$

where V_{SRB} and V_{ANME} represent the volume of individual SRB and ANME cell, respectively. For shell-shaped aggregates of 4 μm in diameter, assuming a single outer layer of SRB cells, and taking the diameters of ANME and SRB cells at 0.5 and 0.4 μm, respectively, the flux ratio of methane to sulfate uptake is 1.0 (see table S1).

Table S1. Consortia size, cell counts, and flux ratio of methane to sulfate uptake ($J_{\mathrm{CH_4}} : J_{\mathrm{SO_4^{2-}}}$) in shell-shaped aggregates with an inner core of ANME cells and a single outer layer of SRB cells.

Consortia diameter (μm)	ANME cell count [(a)]	SRB cell count [(a)]	$J_{\mathrm{CH_4}} : J_{\mathrm{SO_4^{2-}}}$ [(b)]
3	63	189	1.54
4	194	361	0.95
5	438	589	0.69

Note:

(a) Cell counts are calculated by assuming that cells are densely packed and account for 74% of available volumes.

(b) Mass flux ratio is calculated according to equation S4.

S5. Chemical Conditions in Laboratory Bioreactors

Table S2. Sample source, temperature (T, ºC), pH, and concentrations (in mM) of methane, sulfate, dissolved inorganic carbon (DIC), and sulfide in laboratory incubation experiments.

Sample source	T	pH	Methane	Sulfate	DIC	Sulfide	Ref
Eckernförde Bay	15	7.5	1.3	4 (low); 21 (high)	3.8 to 6.9	0.4 (low); 3.7 (high)	[7]
Hydrate Ridge, NE Pacific	4 -16	7.4-7.5	1.4 (low); 14-16 (high)	28	30	0.5-1.0	[8]
Hydrate Ridge, NE Pacific	12	7.5	21	28	30	~0	[6]
Captain Aryutinov Mud Volcano	10	6.8-7.0	3 (low); 90 (high)	10	30	~0	[9]
Captain Aryutinov Mud Volcano	4, 15, 25	7.0	Up to 90 mM	10	30	~0	[10]
Lake Grevelingen	4, 15, 25	7.0	1.7	10	30	~0	[11]
Eckernförde Bay (Baltic Sea)	10-30	6.0-8.5	1.1	19	~0	~0	[12]
Monterey Canyon, Monterey Bay	5	7.0-7.5	1.5	28	~1	~0	[13]

S6. ATP Yields

We predicted that ANME and SRB achieve modest yields, 0.23 – 0.24 mol ATP per mol methane or sulfate. These values are at the lower end of the spectrum compared to other microbial systems, including methanogenesis and sulfate reduction [14]. Among methanogens, only the acetoclastic *Methanosaeta* has a comparable yield of 0.3±0.1 ATP per methane molecule. In contrast, *Methanosarcina* produces 0.6±0.2 ATP during acetoclastic methanogenesis and 1.3±0.2 ATP during hydrogenotrophic methanogenesis. Other hydrogenotrophic methanogens generate around 0.6±0.3 ATP per methane molecule. By comparison, H_2- and acetate-oxidizing SRB yield about 1.5 and 1.0 ATP per sulfate anion, respectively.

S7. References

1. Kojima H et al. A reversed genetic approach reveals the coenzyme specificity and other catalytic properties of three enzymes putatively involved in anaerobic oxidation of methane with sulfate. *Environ Microbiol* 2014;**16**:3431–3442. https://doi.org/https://doi.org/10.1111/1462-2920.12475

2. Scheller S et al. The key nickel enzyme of methanogenesis catalyses the anaerobic oxidation of methane. *Nature* 2010;**465**:606–608. https://doi.org/http://www.nature.com/nature/journal/v465/n7298/suppinfo/nature09015_S1.html

3. Jin Q, Bethke CM. The thermodynamics and kinetics of microbial metabolism. *Am J Sci* 2007;**307**. https://doi.org/10.2475/04.2007.01

4. Fiksen Ø, Follows MJ, Aksnes DL. Trait-based models of nutrient uptake in microbes extend the Michaelis-Menten framework. *Limnol Oceanogr* 2013;**58**:193–202. https://doi.org/10.4319/lo.2013.58.1.0193

5. Knittel K, Boetius A. Anaerobic oxidation of methane: progress with an unknown process. *Annu Rev Microbiol* 2009;**63**:311–334. https://doi.org/10.1146/annurev.micro.61.080706.093130

6. Nauhaus K et al. In vitro cell growth of marine archaeal-bacterial consortia during anaerobic oxidation of methane with sulfate. *Environ Microbiol* 2007;**9**:187–196. https://doi.org/10.1111/j.1462-2920.2006.01127.x

7. Timmers PHA et al. Growth and activity of ANME clades with different sulfate and sulfide concentrations in the presence of methane. *Front Microbiol* 2015;**6**. https://doi.org/10.3389/fmicb.2015.00988

8. Nauhaus K et al. In vitro demonstration of anaerobic oxidation of methane coupled to sulphate reduction in sediment from a marine gas hydrate area. *Environ Microbiol* 2002;**4**:296–305.

9. Zhang Y et al. Stimulation of in vitro anaerobic oxidation of methane rate in a continuous high-pressure bioreactor. *Bioresour Technol* 2010;**101**:3132–3138. https://doi.org/https://doi.org/10.1016/j.biortech.2009.11.103

10. Bhattarai S, Zhang Y, Lens PNL. Effect of pressure and temperature on anaerobic methanotrophic activities of a highly enriched ANME-2a community. *Environmental Science and Pollution Research* 2018;**25**. https://doi.org/10.1007/s11356-018-2573-2

11. Cassarini C, Zhang Y, Lens PNL. Pressure selects dominant anaerobic methanotrophic phylotype and sulfate reducing bacteria in coastal marine Lake Grevelingen sediment. *Front Environ Sci* 2019;**6**. https://doi.org/10.3389/fenvs.2018.00162

12. Meulepas RJW et al. Effect of Environmental Conditions on Sulfate Reduction with Methane as Electron Donor by an Eckernförde Bay Enrichment. *Environ Sci Technol* 2009;**43**:6553–6559. https://doi.org/10.1021/es900633c

13. Girguis PR, Cozen AE, DeLong EF. Growth and Population Dynamics of Anaerobic Methane-Oxidizing Archaea and Sulfate-Reducing Bacteria in a Continuous-Flow Bioreactor. *Appl Environ Microbiol* 2005;**71**:3725–3733. https://doi.org/10.1128/aem.71.7.3725-3733.2005

14. Jin Q. Energy conservation of anaerobic respiration. *Am J Sci* 2012;**312**:573–628. https://doi.org/10.2475/06.2012.01
